# Two phase I/II clinical trials for the treatment of urinary incontinence with autologous mesenchymal stem cells

**DOI:** 10.1002/sctm.19-0431

**Published:** 2020-08-31

**Authors:** Mariano Garcia‐Arranz, Sergio Alonso‐Gregorio, Pamela Fontana‐Portella, Elena Bravo, Jesus Diez Sebastian, María Eugenia Fernandez‐Santos, Damian Garcia‐Olmo

**Affiliations:** ^1^ New Therapies Laboratory Health Research Institute‐Fundación Jiménez Díaz University Hospital (IIS‐FJD) Madrid Spain; ^2^ Surgery Department Autonoma University of Madrid Madrid Spain; ^3^ Department of Urology Hospital De la Cruz Roja San José y Santa Adela Madrid Spain; ^4^ Department of Urology La Paz University Hospital Madrid Spain; ^5^ Department of Plastic Surgery La Paz University Hospital Madrid Spain; ^6^ Statistics Department La Paz University Hospital Madrid Spain; ^7^ GMP‐Cell Production Unit Instituto de Investigación Sanitaria Gregorio Marañón (IiSGM) Madrid Spain

**Keywords:** adipose‐derived stem cells, cellular therapy, male/female urinary incontinence, phase IIa clinical trial

## Abstract

We evaluated the safety and feasibility of adipose‐derived mesenchymal stem cells to treat endoscopically urinary incontinence after radical prostatectomy in men or female stress urinary. We designed two prospective, nonrandomized phase I‐IIa clinical trials of urinary incontinence involving 9 men (8 treated) and 10 women to test the feasibility and safety of autologous mesenchymal stem cells for this use. Cells were obtained from liposuction containing 150 to 200 g of fat performed on every patient. After 4 to 6 weeks and under sedation, endoscopic intraurethral injection of the cells was performed. On each visit (baseline, 1, 3, 6, and 12 months), clinical parameters were measured, and blood samples, urine culture, and uroflowmetry were performed. Every patient underwent an urethrocystoscopy and urodynamic studies on the first and last visit. Data from pad test, quality‐of‐life and incontinence questionnaires, and pads used per day were collected at every visit. Statistical analysis was done by Wilcoxon signed‐rank test. No adverse effects were observed. Three men (37.5%) and five women (50%) showed an objective improvement of >50% (*P* < .05) and a subjective improvement of 70% to 80% from baseline. In conclusion, intraurethral application of stem cells derived from adipose tissue is a safe and feasible procedure to treat urinary incontinence after radical prostatectomy or in female stress urinary incontinence. A statistically significant difference was obtained for pad‐test improvement in 3/8 men and 5/10 women. Our results encourage studies to confirm safety and to analyze efficacy.

## Graphical abstract

We evaluated the safety and feasibility of adipose‐derived mesenchymal stem cells (MSCs) to treat endoscopically urinary incontinence after radical prostatectomy in men or women stress urinary. Our conclusion is intraurethral application of MSCs are a safe and feasible procedure to treat urinary incontinence. A statistically significant difference was obtained for pad‐test improvement in 3/8 men and 5/10 women.

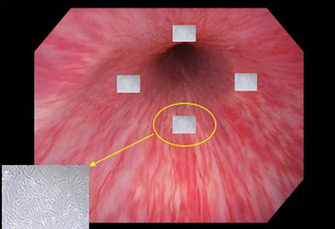

**Lessons learned**

• This safety clinical trial made use of mesenchymal stem cells for the treatment of urinary incontinence.• Intraurethral application of stem cells derived from adipose tissue is a safe and feasible procedure to treat urinary incontinence.

**Significance statement**

This article reports the results of two clinical trials that studied safety of the treatment of urinary incontinence in men and women through the use of mesenchymal stem cells derived from adipose tissue obtained from the patient or cultured ex vivo.

## 1 INTRODUCTION

The International Continence Society defines urinary incontinence as “any involuntary loss of urine that is a social or hygienic problem.” Following benign prostatic hyperplasia surgery, a mean of 0.5% to 2% of male patients develop incontinence,^1^, ^2^ and after radical prostatectomy, this figure ranges from 5% to 60%.^3^ Prostate cancer is the most frequently diagnosed type of cancer in men.^4^

Female stress urinary incontinence (SUI) is the involuntary leakage of urine during events that result in increased abdominal pressure in the absence of a bladder contraction due to effort or exertion or on sneezing or coughing.^5^ In 2017, estimates of its prevalence in the female population range from 10% to 40%.^6^, ^7^ SUI is associated with significant impairment in quality of life and has a significant socioeconomic impact, with costs steadily increasing as the population ages.^8^, ^9^

After radical prostatectomy, urinary sphincter injury is thought to be the main cause of urinary incontinence, as supported by urodynamic studies.^10^, ^11^ Many therapeutic options in male and female urinary incontinence have been used for treatment, with varying degrees of success; these include pelvic floor rehabilitation, bulking agents, slings, and artificial urinary sphincters.^12^, ^13^, ^14^ The cost of the disease borne by health systems is substantial.^15^

New approaches are being considered to improve the treatment, but its development is still uncertain (eg, 3D bioprinted muscle^16^ or tissue engineering^17^). Mesenchymal stem cells (MSCs) have demonstrated, in experimental models, safety and efficacy against urinary incontinence.^18^ But not only MSCs have demonstrated their efficiency in experimental models; in recent years, the proteins secreted by MSCs are generating interesting expectations for the treatment of urinary incontinence.^19^, ^20^ Numerous safety clinical trials have also been developed to analyze the safety of different MSCs for the treatment of urinary incontinence^21^; unfortunately, there are many differences between cellular origin, application system, and follow‐up.

The ability of adult MSCs from adipose tissue, called adipose‐derived stem cells (ASCs), to differentiate into several cell lines has been widely described.^22^ Zuk et al demonstrated the capacity for myocyte differentiation in vitro when cultured next to myoblasts,^23^ and an in vivo experiment in a model of ischemic muscular injury showed ASCs in 20% of myotubes in repair, thereby confirming that ASCs can participate in muscle repair if they are in a “myogenic environment.”^24^ Only two papers have been published on ASCs for urinary incontinence: one used stromal vascular fraction, and the other is a pilot study with ASC cultures combined with bovine collagen in female patients.^25^, ^26^

We present the results of two phase I‐IIa trials to be registered at ClinicalTrials.gov in which autologous ASCs are used for urinary incontinence treatment after radical prostatectomy, and the first one for female SUI.

## 2 MATERIALS AND METHODS

Both trials were approved by the Spanish Agency for Medicines and Health Products (AEMPS) and the Ethics Committee of La Paz Hospital (CEIC) and were registered at ClinicalTrials.gov (NCT01799694 and NCT01804153). All patients signed the informed consent form.

**FIGURE 1:**
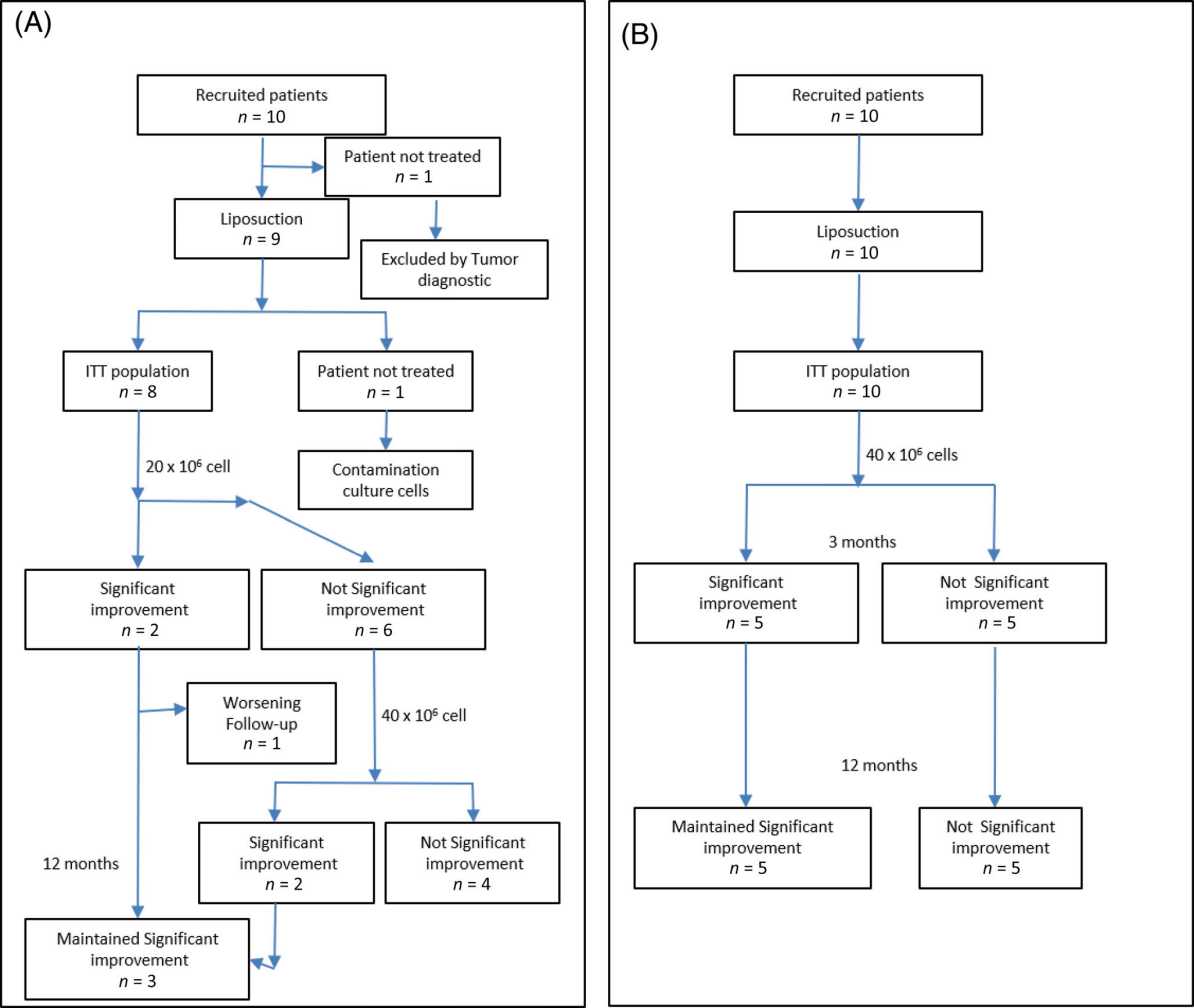
Flowchart. A, The men's clinical trial, in which nine patients were recruited; before cell implant, one patient was excluded because of tumor diagnosis. No adverse events were observed. Significant improvement was achieved by three patients. B, The women's clinical trial, in which 10 patients were recruited; significant improvement was achieved by 5 patients. No adverse events were observed. ITT, intention‐to‐treat

In both, the main objective was to assess the feasibility of the process and the safety of the cell therapy, and the secondary objective was to obtain preliminary results about therapeutic efficacy. We designed two clinical trials in which a total of 10 patients per trial would be recruited. All patients presented urinary incontinence after radical prostatectomy in the men's trial and SUI in the women's trial, and after conservative treatment had failed (Figure 1).

The mean age of the patients enrolled in the men's trial was 67.6 years, with an SD of 5.2; likewise, the incontinence evolution time was 60.5 months, with an SD of 24.5.

The mean age of the patients enrolled in the women's trial was 56.8 years, with an SD of 9.00; likewise, the incontinence evolution time was at least 12 months.

This study was designed to evaluate the feasibility and safety of ASC use and to produce preliminary efficiency results obtained using an independent evaluator. Men were enrolled from May 2011 to February 2012 and women were enrolled from September 2012 to April 2014, according to the inclusion and exclusion criteria (Table 1).

**TABLE 1:** Inclusion and exclusion criteria

*Inclusion criteria—men tria*l
Signed informed consent.
Men over 18 years old. Good general state of health according to the findings of the clinical history and the physical examination.
Prostate cancer‐diagnosed subjects via a biopsy and having had a radical surgery with a healing purpose in the previous 18 months.
Having urinary incontinence after the surgery.
Failure in any previous conservative treatment.
*Inclusion criteria—women trial*
Signed informed consent.
Good general state of health according to the findings of the clinical history and the physical examination.
Postmenopausal or over 18‐years‐old women taking highly effective contraceptives following the ICH (M3) EMA guide.
Women having rejected de‐rehabilitation treatment or in whom the treatment had failed.
Genuine or combined stress urinary incontinence diagnosed with at least 1 year of evolution.
*Exclusion criteria*
Adjuvant therapy.
Prostate‐specific antigen (PSA) ≥0.2 ng/dL after surgery. (Only men's trial)
Present any sign or symptom that could indicate cancer progression. (Only men's trial)
Present bladder outlet obstruction (means by uroflowmetry and urethrocystoscopy).
Active urine infection.
Alcohol or another addictive substances abuse during the 6 months before inclusion.
Presenting any other malignant neoplasia unless it is a basocellular or a skin epidermoide carcinoma or presents antecedents of malignant tumors, unless they are in a remission phase for the previous 5 years.
Cardiopulmonar illness, that by investigator decision, is instable or severe enough as to warrant patient exclusion.
Medical or psychiatric illness, that in the investigators opinion, could imply warrant patient exclusion.
Subjects with congenital or acquired immunodeficiencies, hepatitis B and/or C hepatitis, tuberculosis, or Treponema infection diagnosed at the moment of inclusion.
Pregnant or lactating women. (Only women's trial)
Anesthetic allergy.
Major surgery or severe traumatism within over 6 months prior.
Administration of any drug under experimentation in the present or 3 months before recruitment.

Screening procedures included complete medical history, routine laboratory analysis, urodynamic and uroflowmetry analysis, urethrocystoscopy, a pad test, and quality‐of‐life surveys (The Short Form (36) Health Survey, SF‐36 and International Consultation on Incontinence Questionnaire, ICIQ‐SF).

All patients completed a questionnaire before cell implantation and on every visit as appears in the protocol including complete medical history, biochemistry and hematologic analysis, urodynamic assays, and urethrocystoscopy; Spanish regulatory authorities previously approved both protocols. Patients were diagnosed with urinary incontinence by means of a urodynamic study; in addition, all patients underwent a urethrocystoscopy to rule out urethral abnormalities or abnormalities of vesicourethral anastomosis. Both tests were repeated at the end of the trial.

Liposuction was performed in the plastic surgery department under local sedation. An incision of less than 5 mm was made in the skin and a small‐drilled needle was inserted into the subcutaneous tissue. In all cases, we obtained 150 to 200 cc of adipose tissue.

The treatment began, in the men's clinical trial, with an initial implantation of 20 × 10^6^ ASCs followed by a clinical evaluation after 3 months, and if significant improvement in incontinence was not achieved, a second implantation of 40 × 10^6^ ASCs was performed. In the women's clinical trial, only a cell implantation of 40 × 10^6^ ASCs was performed with the same follow‐up.

### 2.1 ASC preparation

The isolation of ASCs from lipoaspirate has been described previously.^27^ We used MSCs derived from subcutaneous adipose tissue obtained by liposuction and processed by the Gregorio Marañón Hospital (Madrid, Spain; manufacturer authorization no: AEMPS‐20090211‐TA and ES/125I/18, according to the PEI 04‐031; Product in Clinical Research) and according to Spanish and European legislation (ASC production is only permitted in good manufacturing practice conditions). ASCs were obtained exclusively by collagenase digestion and culture with Dulbecco's modified Eagle's medium supplemented with 10% fetal bovine serum, after washing extensively and removing cells attached to the plastic; Before applying to patients according to the EMEACHMP4108692006 cell therapy guide, cells were characterized as mesenchymal stem cell: cell differentiation to osteoblast, chondrocytes and adipocytes, and flow cytometry with positive (CD27, CD44, CD90, and CD105) and negative markers (CD34, CD45, and CD73) (Supplementary Data). Cultivation and expansion of cells continued in an authorized procedure until the required number of cells for implantation (dose) was obtained. For quality‐control and logistical reasons, the doses of cells were cryopreserved in liquid N_2_ (30% cell death: producer data). At least 1 week before the date of implantation, the cells were thawed and cultured. For administration, the cells were suspended in a sterile lactated Ringer's solution with 1% human albumin at 1 × 10^7^ cells/mL. Samples were taken before release to examine viability, DNA stability, and pathogen controls (analysis performed by the producer). For implantation, 20 × 10^6^ ASCs were prepared and 40 × 10^6^ were cryopreserved for each male patient, in case they need a second dose, and 40 × 10^6^ ASCs were prepared for each female patient.

### 2.2 Treatment

In both trials, under direct vision and with the patient under sedation and in the lithotomy position, cell implantation was performed in the area close to the bladder neck and along the external sphincter using a compact cystoscope (17 Ch) and an endoscopic needle (7 Ch) for cell injection. A random distribution of cellular material was made, injecting volumes of 0.2 to 0.3 mL of ASCs at a depth of less than 0.5 mm; 7 to 8 injections (volume: 2 mL) were performed in all cases in the urinary sphincter of men and in women from the neck to the middle urethra.

### 2.3 Follow‐up

The feasibility of the process was assessed based on the absence of problems during sample collection, cell culture, and cell implantation, although in one case, the tissue from the liposuction was contaminated and liposuction had to be repeated because the fungal contamination could not be eliminated during cell culture.

Safety was assessed in terms of the incidence of adverse events and serious adverse events. Patients were monitored for adverse events at each study visit (implant, 4, 12, 24 weeks, and 1 year) and for any other observation that could alert of a possible abnormal/deleterious impact of ASCs. During follow‐up, we analyzed changes in medical history, blood pressure, heart rate, temperature, systemic parameters (biochemical and hematological features), and local parameters (urine culture, uroflowmetry, cystoscope, and urodynamic evaluation). We also collected SF‐36 and ICIQ‐SF surveys.

Clinical response was defined as an objective improvement in urine leakage of more than 50% relative to baseline values as quantified by the pad test.

### 2.4 Statistical analysis

The database was analyzed using the SPSS v.11.5 program (IBM, Armonk, New York). *P* values of <.05 were considered statistically significant. Values are shown in numbers and percentages. Quantitative values were compared by performing a temporal evolution study, and a before‐after paired test was compared using a nonparametric Wilcoxon test (Wilcoxon signed‐rank test).

## 3 RESULTS

In the men's trial, 10 patients were recruited, one of whom was diagnosed with a pancreatic tumor after liposuction and was excluded before cellular treatment. In another case, the cells were contaminated and liposuction had to be repeated, which left us with eight patients finally treated with cell therapy.

In the women's trial, 10 patients were recruited, all were treated, and all the follow‐up was carried out without deviations from the protocol.

In all cases, liposuction and cell culture were performed without any incident except for one case of fungal contamination in the men's trial, which led to a repeat liposuction. The patients may have occasional local pain and a slight hematoma in the abdominal region. In all cases, endoscopic injection was performed without complications. Most patients complained of dysuria for 2 to 3 days. The trial showed that isolation, expansion, and implantation of cells are feasible.

From a safety point of view, the cell therapy had no adverse effects on any patients in any trial, neither from a clinical nor from an analytic perspective. The data recorded did not reveal any adverse events (AEs) as concerns all of the variables collected.

In the men's trial, none of the patients suffered a biochemical relapse of their prostate cancer (prostate‐specific antigen [PSA] value) during the 12‐ to 36‐month follow‐up. Two of the eight patients received only the first injection of 20 × 10^6^ ASCs, and the other six received a second injection of 40 × 10^6^ ASCs after 3 months (Figure 1A). Cases 5 and 9 (2/8) showed an objective clinical improvement of more than 50% after 3 months of follow‐up after 20 × 10^6^ ASC treatment; this improvement, with no AEs related to cell therapy, was confirmed by the absence of abnormalities in the uroflowmetry test or pad test performed during each visit and with a urethrocystoscopy at final evaluation (Table 2). Case 9 worsened after 6 months and returned to baseline by month 9. Case 5 maintained this improved state after 12 months of follow‐up and went from 16 g of urine leakage to 3 g at the end of the trial (Tables 2, 3).

**TABLE 2:** Urodynamics and voiding evaluation

Men	Urethral profile		Cough leakage		Urodynamics	
	Before implant	End line	Before implant	End line	Before ‐implant	End line
Case 1	<5	<5	Positive	Positive	UI	UI
Case 2	14	59	Positive	Negative^a^	UI	No UI^a^
Case 3	8	12	Positive	Positive	UI	UI
Case 4	12	12	Positive	Positive	UI	UI
Case 5	18	10	Positive	Negative^a^	UI	UI^a,b^
Case 6	Excluded					
Case 7	26	20	Positive	Positive	UI	UI
Case 8	13	62	Positive	Negative^a^	UI	No UI^a^
Case 9	67	12	Positive	Positive	UI	UI

Women	Urethral profile		Cough leakage		Urodynamics	
	Before implant	End line	Before implant	End line	Before implant	End line
Case 1	22	16	Positive	Positive	UI	UI
Case 2	30	35	Positive	Positive	UI	UI
Case 3	No obtained	40	Positive	Negative^a^	UI	UI^a,^ ^b^
Case 4	21	33	Positive	Positive	UI	UI
Case 5	74	91	Positive	Negative^a^	UI	No UI^a^
Case 6	24	23	Positive	Negative^a^	UI	No UI^a^
Case 7	61	84	Positive	Negative^a^	UI	No UI^a^
Case 8	61	36	Positive	Negative^a^	UI	No UI^a^
Case 9	43	35	Positive	Negative^a^	UI	No UI^a^
Case 10	31	27	Positive	Positive	UI	UI

*Note:* Urethral profile measured in CmH_2_O.

Abbreviation: UI, urinary incontinence.

^a^ Patients with good clinical response.

^b^ Improvement.

**TABLE 3:** Urinary incontinence measurement (pad test in grams)

Men	20 ASCs million ASCs					40 ASCs million ASCs				
(*P* < .05)	Before implant	Visit 1	Visit 2	Visit 3	End line	Before implant	Visit 1	Visit 2	Visit 3	End line
Case 1	40	35	38	44	40	40	62	70	44	54
Case 2^a^	41	30	20	24	41	41^a^	30^a^	20^a^	2^a^	9^a^
Case 3	169	55	98	76	169	169	120	90	149	130
Case 4	42	32	19	31	42	42	36	34	36	44
Case 5^a^	16^a^	12^a^	12^a^	10^a^	3^a^	Not applicable				
Case 6						Not applicable (patient excluded prior to cell implantation)				
Case 7	147	197	178	100	147	147	138	114	230	138
Case 8^a^	287	295	246	218	287	287^a^	83^a^	110^a^	118^a^	123^a^
Case 9	4	1	1	2	3	Not applicable				

Women						40 ASCs million ASCs				
(*P* < .05)						Before implant	Visit 1	Visit 2	Visit 3	End line
Case 1						11	8	10	3	21
Case 2						7	2	23	20	12
Case 3						1	1	1	0	2
Case 4						14	4	2	7	13
Case 5^a^						22^a^	8^a^	10^a^	11^a^	6^a^
Case 6^a^						22^a^	15^a^	12^a^	2^a^	1^a^
Case 7^a^						17^a^	4^a^	5^a^	6^a^	0^a^
Case 8^a^						14^a^	48^a^	14^a^	6^a^	5^a^
Case 9^a^						18^a^	10^a^	14^a^	6^a^	0^a^
Case 10						96	58	120	141	69

Abbreviation: ASCs, adipose‐derived stem cells.

^a^ Patients with positive evolution.

Cases 2 and 8 (2/6) were treated with a second cell injection of 40 × 10^6^ ASCs and showed an objective clinical improvement of more than 50%, which was maintained over 12 months of follow‐up. This improvement, with no AEs related to cell therapy, was confirmed by the absence of abnormalities in the uroflowmetry test and pad test performed during each visit and with a urethrocystoscopy at final evaluation (Table 2). Case 2 went from 41 g of urine leakage to 9 g at final evaluation, and case 8 progressed from 287 to 123 g (Table 3).

In the women's trial, all patients received only an injection of 40 × 10^6^ ASCs (Figure 1B). Cases 4‐8 (5/10) showed an objective clinical improvement of more than 50% after 3 months of follow‐up after 40 × 10^6^ ASC treatment; this improvement, with no AEs, was confirmed by the absence of abnormalities in the uroflowmetry test and pad test performed during each visit and with an urethrocystoscopy at final evaluation (Table 2).

Cases 5‐9 (5/10) showed an objective clinical improvement at final evaluation. Four patients were continent and one showed an objective clinical improvement of more than 50% a year after cell implantation. Case 10 passed from 96 pads before cell implant to 69 pads. And cases 3 and 4 showed a very slight improvement. In cases 1 and 2, we consider that there was a bulking effect due to the observed evolution (Table 3).

In summary, in the men's and women's trials, three (37.5%) and five (50%) patients, respectively, showed an objective clinical improvement of more than 50%, constituting a statistically significant difference (*P* < .05). This improvement was maintained over time and showed in pad test and urodynamic evaluation.

We used the SF‐36 and ICIQ‐SF for the quality‐of‐life analysis and were unable to prove any statistical differences during follow‐up in any trials.

## 4 DISCUSSION

Surgical treatment is the gold‐standard therapy for urinary incontinence, when noninvasive therapies have failed and short‐term success has been achieved with injectable bulking agents.^28^, ^29^ The standard treatment for urinary incontinence in men is a conservative treatment during the first year after surgery, according to our experience and the recommendations of Kadoto et al,^30^ and a second treatment with an artificial urinary sphincter. The standard treatment in women is tension‐free suburethral mesh if the treatment by rehabilitation of the pelvic floor has failed. The success rate for those procedures in men is at least 80%, the complication rate is <10%, and the patient satisfaction rate is around 75% to 95%. The success rate for the procedure in women is more than 80% to 90% after more than 5 years of follow‐up. However, these procedures have certain complications.^31^ Complications, high cost, and not 100% long‐term efficacy force us to seek alternative treatments for SUI.^32^, ^33^ Stem cell therapy has been investigated in different clinical applications.^34^, ^35^, ^36^, ^37^, ^38^ Taking advantage of its capacity to induce tissue regeneration, stem cell treatment may be a promising strategy to overcome the current treatments for SUI.^39^

In these studies, we used autologous cells to avoid the risk of rejection. Another important aspect of cellular therapy is the progressive normalization of the associated costs, thus making it an increasingly cost‐effective option.

We believe that the low risk generated during outpatient liposuction followed by implantation of cells by endoscopic injection in the sphincter while the patient is under sedation, has advantages over traditional treatments. Moreover, the cost of both procedures (including liposuction and cell implant) is less than that of conventional treatments, mainly because the treatment occupies a short surgical room time and the patient does not need hospitalization. And this will be lower in the future if we can carry out allogenic treatments.

Sample collection, processing, and cell cultivation were performed under protocols designed by the research team and the production laboratory and were approved and validated by CEIC and AEMPS. It is noteworthy that cell expansion is performed in adherence of legislation and in licensed facilities, thus guaranteeing that the product administered to patients is homogeneous. In addition, unlike several previously described endoscopic treatments (bulking agents), we observed no cases of urethral structure and/or rigidity in the injection zones, nor did we find any alteration of voiding quality or worsening of incontinence as evidenced by uroflowmetry, urodynamic study, and pad tests performed on every visit. Furthermore, on the last visit, an urethrocystoscopy was performed on all patients to ensure the safety and effectiveness of intraurethral injections compared with the baseline.

Multiple parameters were collected for every patient before cell implantation and during every visit. We found no evidence of a systemic effect of cell therapy. More importantly, we observed no PSA‐level alterations in either the follow‐up of any patient or in the subsequent routine follow‐up (>3 years after cell therapy) in the men's trial. We can thus state that cell therapy, when administered in selected patients who meet disease‐free criteria, does not interfere with prostate cancer evolution, at least in our series of patients. Oncologic safety is a major issue in cell therapy, and several studies support this oncologic safety in time and support our clinical findings obtained over the short follow‐up involved in this study.^40^, ^41^, ^42^

The number of patients included does not allow us to make any statements on the effectiveness of the treatment. Probably the best way to evaluate urinary incontinence is the pad test and assessment of urine leakage through quality‐of‐life questionnaires.^43^, ^44^, ^45^, ^46^ We used two questionnaires and did not find any statistical differences with these tools, likely because they were not the best surveys and no patient was cured in the men's trial. However, we think that the SF‐36 questionnaire is not the most appropriate to analyze the quality of life of patients with incontinence, and for future trials, we propose using the Incontinence Quality of Life Questionnaires (IQOL) Test or King's Health Questionnaire.^47^ From an objective perspective, we used the number of pads per day, the 24‐hour pad test, and urodynamic studies (Tables 2 and 3). During follow‐up, we defined two time points for evaluation of therapeutic efficacy (3 and 12 months), making this decision in light of published accounts stating that, for endoscopic bulking agent treatments, initial response was lost after the disappearance of the bulking effect during the first 3 months.^14^, ^28^, ^48^

The decision to schedule two cell doses was informed by the current lack of knowledge on optimal implanted cell dose.^49^, ^50^ Because of this and the fact that the maximum dose allowed by the AEMPS at the time of this trial was 40 × 10^6^ ASCs, we decided on a dosage escalation program that called for 20 × 10^6^ in the first injection and 40 × 10^6^ in the second injection (Figure 1) in the first clinical trial (men); owing to the excellent safety profile and to reduce costs, we decided to use only the highest dose in the second clinical trial (women). In addition, magnetic resonance imaging has been shown to be a good tool to evaluate the sphincter; it has not been considered in this phase I‐IIa trial, but it could be useful in a phase II trial.

As concerns the clinical improvement evidenced in 37.5% of the men (3/8) and 50% of the women (5/10) and maintained after more than 12 to 15 months of follow‐up, it is important to highlight that in the urodynamic study of these patients, no urinary incontinence was found. It should be noted that these patients (>50% decrease on pad test; *P* < .05) reported a subjective improvement of 70% to 80%, and no surgical procedure to correct residual urinary incontinence was considered by the patients. The variability of the pad test depends on several factors such as oral intake, weather, exercise, and so on, thus explaining the positive and negative variation found within a given patient. It is noteworthy that only the differences obtained for these patients of urine‐leakage improvement were statistically significant (*P* < .05), so the other nonsignificant difference obtained on improvement or worsening must be secondary to the intrinsic variability of the pad test. Anyway, in all cases, these patients are the patients that showed improvement in the urodynamic test.

For us, is difficult to know if the difference of results between both trials is due to the cellular dose, to the type of incontinence, or to other factors (sex of the patients, age, etc.); it is important to consider that we are working a medicine live, and its manipulation implies a learning curve. In addition, the average age of the patients included in the men's trial exceeded 60 years, and it is well known that stem cells have decreased abilities with age (aging).^51^

There are many issues surrounding the mechanism by which stem cells act. Stem cells are known for their ability to differentiate into several cell lines, thus giving rise to their theoretical ability to restore damaged tissue. Furthermore, their paracrine effect has become more and more relevant over the last years; ASCs provide soluble mediators like cytokines and growth factors, which have effects upon cytoprotection, angiogenesis, tissue repair, and the normalization of the extracellular matrix and relief from inflammation.^52^ If we can continue in this direction and answer all these questions, we will be poised to offer incontinence therapy that is safe, efficient, effective, minimally invasive, and lasting. According to this idea, in recent years, the secretome of ASCs for the treatment of urinary incontinence has been analyzed, and this way of working could inhibit some of the problems associated with the route of application, cell expansion/dose, and so on; however, despite appearing promising, it is an almost unknown treatment.^53^

Therefore, we can state that ASC therapy for urinary incontinence after radical prostatectomy or female SUI is safe and feasible, although its efficacy is relative, at least with the doses used in our trial and with a limited number of patients. We may conclude that 20 million ASCs are probably insufficient, although some patients had an obvious response; 40 million ASCs might be therapeutic for mild to moderate incontinence and have relative efficacy for moderate or severe incontinence. In any event, more trials to investigate the best cell dose are required.

## 5 CONCLUSION

As our main conclusion, we can state that ASC therapy is a feasible and safe therapy from all points of view for the treatment of urinary incontinence in men and women, a finding that was the main objective of our trial. Both the direct repair effect and the paracrine repair effect of ASCs require further trials to achieve safety and efficacy for stem‐cell therapy.

## CONFLICT OF INTEREST

Mariano Garcia‐Arranz declared inventor or patent holder, consultant/advisory role, and expert testimony for Biosurgery, an educational company. Damian Garcia‐Olmo declared inventor or patent holder and consultant/advisory role for Biosurgery, an educational company, is a member of the advisory board of Tigenix S.A.U., and has received fees from Takeda. The other authors indicated no potential conflicts of interest.

## AUTHOR CONTRIBUTIONS

M.G.‐A., S.A.‐G.: conception and design, financial support, administrative support, collection of data, data analysis and interpretation, manuscript writing, and final approval of manuscript; P.F.‐P.: collection of data, provision of patients, and final approval of manuscript; E.B., M.E.F.‐S.: provision of study material and final approval of manuscript; J.D.S.: data analysis and final approval of manuscript; D.G.‐O.: conception and design, financial support, data analysis and interpretation, and final approval of manuscript.

## Supporting information


**Supplementary Data**: Lipoaspirate characteristics (sex and volume) and tests carried out before the packaging of the final product according to the EMEACHMP4108692006 cell therapy Guide. Up table, Viability, differentiation potential and safety test (mycoplasma, sterility and genetic stability). Down table, flow cytometry analysis.

## Data Availability

The data that support the findings of this study are available on request from the corresponding author.
