# Supplementary material for: Two phase I/II clinical trials for the treatment of urinary incontinence with autologous mesenchymal stem cells
Source: Stem Cells Transl Med. 2020 Aug 31;9(12):1500–8. doi: 10.1002/sctm.19-0431 (PMC7695632; doi:10.1002/sctm.19-0431)
Supplement: Supplementary file 1 — Supplementary Data: Lipoaspirate characteristics (sex and volume) and tests carried out before the packaging of the final product according to the EMEACHMP4108692006 cell therapy Guide. Up table, Viability, differentiation potential and safety test (mycoplasma, sterility and genetic stability). Down table, flow cytometry analysis. [file SCT3-9-1500-s001.pdf]

BATCH OF NCT01799694		URO 1	URO 2	URO 3	URO 4	URO 5	URO 6	URO 7	URO 8	URO 9
GENDER		MALE	MALE	MALE	MALE	MALE	MALE	MALE	MALE	MALE
LIPOASPIRATED VOLUME (ml)		95	180	100	120	100	150	90	150	125
MNCs VIABILITY (%)		80%	89%	95%	85%	91%	90%	89%	83%	78%
ADSCs VIABILITY (%)		94%	96%	90%	84%	80%	93%	91%	86%	83%
FIRST DOSE (106)		20	25	19,5	22	22	Infección pulmonar.	22	22,5	22
SECOND DOSE (106)		45	43	40	42	-		42	42	-
DIFFERENTIATION POTENTIAL	ADIPOCYTES	POSITIVE	POSITIVE	POSITIVE	POSITIVE	POSITIVE	POSITIVE	POSITIVE	POSITIVE	POSITIVE
	OSTEOBLASTS	POSITIVE	POSITIVE	POSITIVE	POSITIVE	POSITIVE	POSITIVE	POSITIVE	POSITIVE	POSITIVE
	CHONDROBLASTS	POSITIVE	POSITIVE	POSITIVE	POSITIVE	POSITIVE	POSITIVE	POSITIVE	POSITIVE	POSITIVE
MYCOPLASMA		NEGATIVE	NEGATIVE	NEGATIVE	NEGATIVE	NEGATIVE	NEGATIVE	NEGATIVE	NEGATIVE	NEGATIVE
STERILITY		STERILE	STERILE	STERILE	STERILE	STERILE	STERILE	STERILE	STERILE	STERILE
GENETIC STABILITY		STABLE	STABLE	STABLE	STABLE	STABLE	STABLE	STABLE	STABLE	STABLE

BATCH OF NCT01804153		URO 10-1	URO 10 2	URO 10-3	URO 10-4	URO 10-5	URO 10-6	URO 10- 7	URO 10-8	URO10-10	URO 10-11
GENDER		FEMALE	FEMALE	FEMALE	FEMALE	FEMALE	FEMALE	FEMALE	FEMALE	FEMALE	FEMALE
LIPOASPIRATED VOLUME (ml)		150	180	350	250	250	205	50	350	125	125
MNCs VIABILITY (%)		70%	80%	90%	88%	78%	85%	91%	92%	93%	81%
ADSCs VIABILITY (%)		96%	90%	88%	89%	92%	91%	86%	95%	90%	94%
INFUSED DOSE (106)		43	42	40	37	40	12,85	34,5	42	42	38
DIFFERENTIATION POTENTIAL	ADIPOCYTES	POSITIVE	POSITIVE	POSITIVE	POSITIVE	POSITIVE	POSITIVE	POSITIVE	POSITIVE	POSITIVE	POSITIVE
	OSTEOBLASTS	POSITIVE	POSITIVE	POSITIVE	POSITIVE	POSITIVE	POSITIVE	POSITIVE	POSITIVE	POSITIVE	POSITIVE
	CHONDROBLASTS	POSITIVE	POSITIVE	POSITIVE	POSITIVE	POSITIVE	POSITIVE	POSITIVE	POSITIVE	POSITIVE	POSITIVE
MYCOPLASMA		NEGATIVE	NEGATIVE	NEGATIVE	NEGATIVE	NEGATIVE	NEGATIVE	NEGATIVE	NEGATIVE	NEGATIVE	NEGATIVE
STERILITY		STERILE	STERILE	STERILE	STERILE	STERILE	STERILE	STERILE	STERILE	STERILE	STERILE
GENETIC STABILITY		STABLE	STABLE	STABLE	STABLE	STABLE	STABLE	STABLE	STABLE	STABLE	STABLE

FLOW CITOMETRY

MALE	CD 45	CD 34	CD 14	CD 19	HLA DR	CD 105	CD 73	CD 90	CD 117	VEGF	CD 133	CD 106	CD 71	CD 271	CD 29	CD 44	CD 31	CD 184
Uro-1	0,19	0,55	0,1	0,81	0,09	92,09	99,93	99,98	0,05	77,17	6,85	71,94	98,06	87,82	99,32	98,3	1,78	72,53
Uro-2	0,15	1,05	0,32	0,75	0,15	99,44	99,99	99,99	0,01	82,46	4,64	77,12	98,86	74,62	99,69	96,99	1,97	74,6
Uro-3	0,39	1,82	0,39	1,08	0,33	99,7	99,96	99,91	0,14	85,17	11,41	28,44	95,04	82,2	98,91	95,75	0,99	75,23
Uro-4	0,24	0,43	0,25	0,49	0,12	97,92	99,96	99,81	0,1	83,76	21,23	65,54	98,02	39,95	99,97	99,87	0,09	64,79
Uro-5	0,28	0,37	0,26	0,14	0,02	94,94	99,99	99,81	0,23	86,73	11,89	32,8	98,93	66,59	99,96	99,94	0,27	81,52
Uro-6	0,25	0,7	0,19	0,17	0,06	97,9	100	90,55	0,26	89,5	9,93	85,02	95,56	59,98	99,96	99,75	0,49	84,56
Uro-7	0,14	0,16	0,08	0,58	0,18	95,85	99,99	99,4	0,12	80,7	9,78	64,89	95,55	32,17	99,99	99,77	0,03	50,72
Uro-8	0,4	0,19	0,06	0,24	0,11	95,95	100	99,7	0,1	82,79	16,43	66,38	97,61	31,36	100	99,77	0,12	65,91
Uro-9	0,11	0,15	0,15	0,35	0,81	94,95	99,96	99,92	0,05	82,92	1,12	91,25	96,01	8,78	99,82	99,13	0,1	95,74
FEMALE																		
URO10-001	0,21	0,75	0,16	1,21	0,44	90,83	99,99	98,65	0,03	70,41	0,58	80,77	97,46	9,2	99,96	99,53	0,2	91,84
URO10-002	1,42	0,87	1,26	1,39	1,5	98,18	99,91	99,6	0,18	79,95	15,47	55,87	99,07	9,66	99,99	99,82	0,19	6,29
URO10-003	0,92	4,93	0,53	1,21	0,8	99,18	99,97	99,91	0,02	79,84	0,84	86	96,13	11,25	99,99	99,83	0,4	65,42
URO10-004	0,68	14,55	0,53	1,22	1,09	99,44	99,99	99,87	0,05	78,01	0,95	84,48	92,58	11,77	99,98	99,72	0,55	49,3
URO10-005	1,19	4,36	0,86	1,2	0,95	99,4	99,9	90,6	0,47	79,81	0,88	47,34	0,62	18,48	99,98	99,85	1,32	93,52
URO10-006	0,36	1,84	0,52	0,57	0,45	95,19	99,99	94,87	0,03	86,03	0,52	86,64	99,84	29,32	99,97	99,81	1,66	63,61
URO10-007	0,26	1,95	0,26	0,22	0,3	98,5	99,99	98,92	0,09	84,7	0,28	85,16	99,79	29,7	100	99,72	0,73	72,25
URO10-008	1,09	1,97	0,34	0,42	0,38	96,96	99,99	98,81	0,2	92,21	0,8	91,84	99,48	4,96	99,99	98,57	1,62	81,78
URO10-010	0,29	0,14	0,24	0,38	0,35	96,78	99,99	99,86	0,07	79,89	0,19	81,62	99,5	60,59	99,98	99,99	0,74	90,17
URO10-011	1,07	0,32	0,92	1,55	1,07	99,9	99,98	99,49	0,27	81,64	3,44	82,49	98,5	99,63	92,33	99,98	0,57	93,26
